# Diastereoselective synthesis of 3,4-dihydro-2*H*-pyran-4-carboxamides through an unusual regiospecific quasi-hydrolysis of a cyano group

**DOI:** 10.3762/bjoc.12.198

**Published:** 2016-09-27

**Authors:** Mikhail Yu Ievlev, Oleg V Ershov, Mikhail Yu Belikov, Angelina G Milovidova, Viktor A Tafeenko, Oleg E Nasakin

**Affiliations:** 1 Department of Organic and Pharmaceutical Chemistry, Ulyanov Chuvash State University, Moskovskiy av. 15, Cheboksary, Russia; 2 Department of General Chemistry, Lomonosov Moscow State University, Leninskie gory 1, Moscow, Russia

**Keywords:** diastereoselectivity, 3,4-dihydro-2*H*-pyran-4-carboxamide, nitriles, pyran, quasi-hydrolysis

## Abstract

An efficient diastereoselective approach for the synthesis of functionalized 3,4-dihydro-2*H*-pyran-4-carboxamides with variable frame was developed based on the reaction of available 4-oxoalkane-1,1,2,2-tetracarbonitriles (adducts of TCNE and ketones) with aldehydes in an acidic media. An unusual process of quasi hydrolysis of the cyano group was observed in the course of the described regio- and diastereoselective transformation.

## Introduction

Dihydro- and tetrahydropyran moieties are very important structural fragments in organic synthesis. They are part of many natural compounds, biologically active substances and drugs [1–4]. For instance, Zanamivir and Laninamivir which are recommended for the treatment and prophylaxis of influenza caused by influenza A and B viruses contain a 3,4-dihydro-2*H*-pyran fragment [5]. Moreover, dihydropyrans have been proven to be particularly useful in the preparation of cyclic components of macrocyclic antibiotics [6–7] and as precursors in the synthesis of C-glycosides [8].

**Scheme 1:**
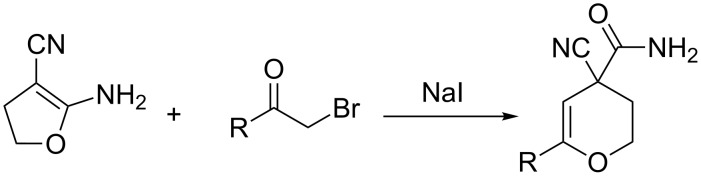
An exclusive approach to 3,4-dihydro-2*H*-pyran-4-carboxamides from non-pyran sources.

Modern and convenient methods for the construction of 3,4-dihydro-2*H*-pyrans are based on the interaction between ylidene derivatives of methylene-active compounds and β-oxo derivatives of acids or ketones [9–11], and also on the reactions of phenols with carbonyl compounds [12]. An interesting example that had been recently described is the involvement of diazolactones in an inverse electron-demand Diels–Alder reaction [13]. At the same time the synthesis of 3,4-dihydro-2*H*-pyrans with a carboxamide group is a not sufficiently explored area. There is only one way to produce 3,4-dihydro-2*H*-pyran-4-carboxamides from substrates containing no pyran ring described in the literature (Scheme 1) [14].

**Scheme 2:**
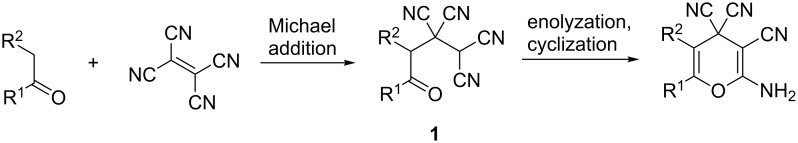
Known approach to pyran derivatives based on ketonitriles **1**.

In view of a directed synthesis of inaccessible heterocyclic molecules, 4-oxoalkane-1,1,2,2-tetracarbonitriles **1** are very promising substrates. They can be easily prepared from an appropriate ketone and TCNE (Scheme 2). Previously we reported about several ways of heterocyclization thereof [15–23], including the formation of pyran derivatives [20–24].

Our recent work should be noted individually, we were able to prepare various functionalized pyrano[3,4-*c*]pyrrole derivatives via diastereoselective cascade reaction [23]. The crucial stage of the described transformation is the formation of a pyran-4-carboxamide intermediate. A trace amount of it was isolated accidentally and we could not repeat this procedure and characterize the compound by spectra.

## Results and Discussion

In continuation of our interest in this area, we focused our attention on the extention of the existing methods for the synthesis of a series of pyran heterocycles. Therefore, we have studied more thoroughly the transformation of ketonitriles **1** in acidic media in the presences of aldehydes. We had found that 3,3,4-tricyano-3,4-dihydro-2*H*-pyran-4-carboxamides **2** nevertheless could be obtained in good yields (57–69%) by the action of hydrochloric acid. A prominent feature of the reaction that has been developed is the possibility to vary substituents at the 2-, 5- and 6-positions of the pyran cycle, using alkyl and aryl moieties to design the rare pyran-4-carboxamide molecules. Moreover, during the regio- and diastereoselective transformation a quasi-hydrolysis of only one of the cyano groups had occurred. As a result only one diastereomer of 3,4-dihydro-2*H*-pyran-4-carboxamides **2** was obtained (Table 1).

**Table 1:** Synthesis of 3,3,4-tricyano-3,4-dihydro-2*H*-pyran-4-carboxamides **2**.

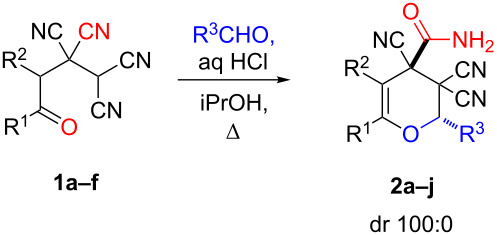				

Entry	Ketonitrile	Aldehyde	Product	Yield (%)

1	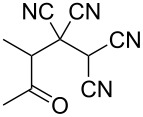 **1a**	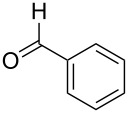	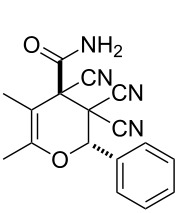 **2a**	57
2	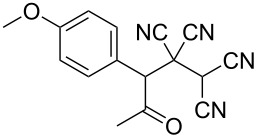 **1b**	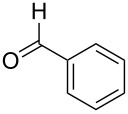	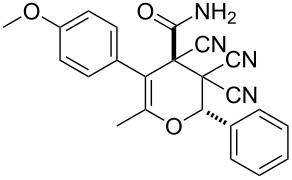 **2b**	59
3	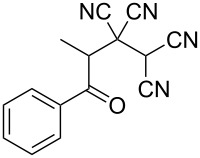 **1c**	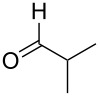	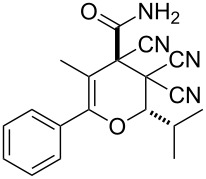 **2c**	64
4	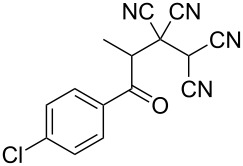 **1d**	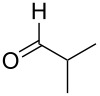	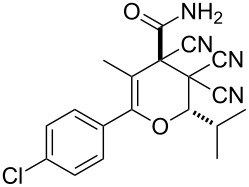 **2d**	61
5	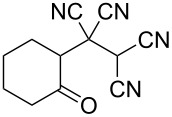 **1e**	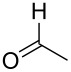	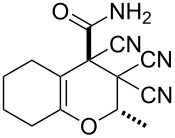 **2e**	66
6	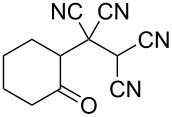 **1e**	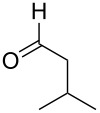	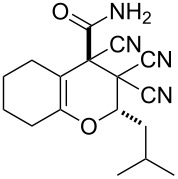 **2f**	64
7	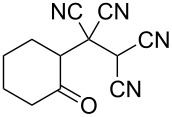 **1e**	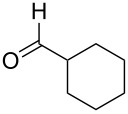	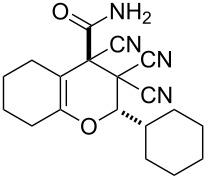 **2g**	61
8	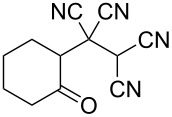 **1e**	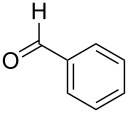	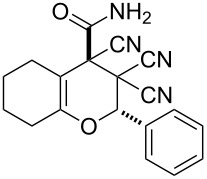 **2h**	69
9	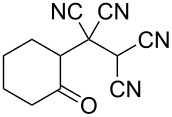 **1e**	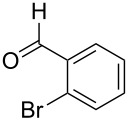	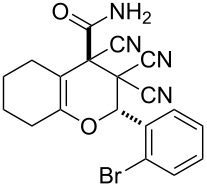 **2i**	67
10	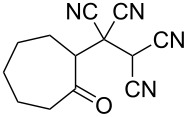 **1f**	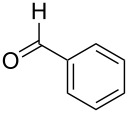	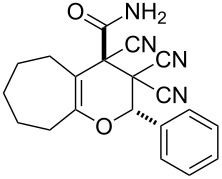 **2j**	59

**Figure 1:**
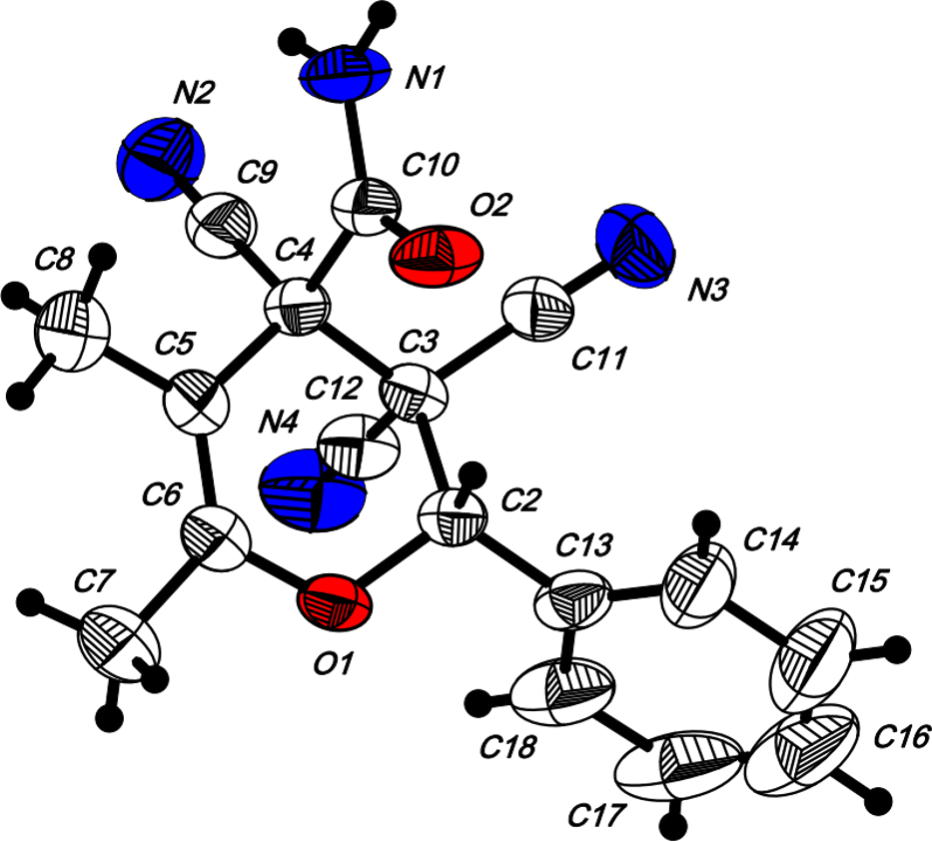
The molecular structure of **2a** with atom-numbering scheme. Displacement ellipsoids are drawn at the 50% probability level and H atoms are drawn as small spheres of arbitrary radii.

The structure of pyrans **2** as well as the *trans* configuration of the substituents at the asymmetric atoms C2 and C4 were established by X-ray diffraction (Figure 1) [25]. The conformation of the six-membered heterocyclic ring is stabilized by an intermolecular hydrogen bond C2–H2···O2. The distance O2···H2 is 2.31 Å. In the crystal, the molecules form centrosymmetric dimers through hydrogen bonding N1–H11···O2 (H1···O2 1.98 Å, angle N1–H1···O2 176°). Structures of compounds **2** are also consistent with IR, ^1^H NMR spectroscopy and mass spectrometry. The IR spectra characterized with the absorption bands of non-conjugated C≡N in the area of 2240–2260 cm^−1^ and two intensive bands of amide N–H bonds in the area of 3150–3450 cm^−1^. The ^1^H NMR spectra of compounds **2** are characterized with two individual downfield signals of amide protons in the area of 8–9 ppm, signals of the OCH fragment in the area of 4.9–6.5 ppm, depending on the substituent R^3^, and the signals of other appropriate alkyl and aryl substituents.

**Scheme 3:**
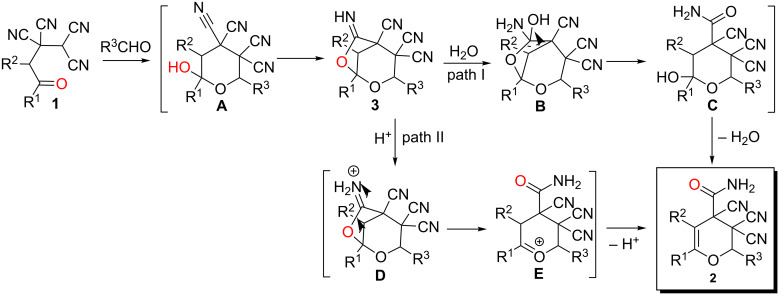
Plausible reaction pathways for 3,4-dihydro-2*H*-pyran-4-carbxamides **2** formation.

There are two cascade mechanisms that could be proposed for the 3,4-dihydro-2*H*-pyran-4-carboxamides **2** formation (Scheme 3).

Both mechanisms apparently start from the interaction of ketonitrile **1** with aldehyde under subsequent formation of the intermediate 2,7-dioxabicyclo[3.2.1]octane derivative **3**. This reaction was separately described by us previously and it is the crucial reason for the diastereoselectivity of the whole transformation [21–22]. Further acid-catalyzed addition of water to the imino group (**B**) is probably accompanied with decyclization (**C**) and dehydratation processes forming compound **2** (Scheme 3, path I). However, according to the literature, in most cases, the addition of water to the similar cyclic iminoethers leads to hydrolysis and formation of lactones [26–27]. Another probable pathway involves the protonation of the imino group nitrogen atom to form an iminium salt (**D**) and subsequent decyclization of iminolactone ring (**E**) without participation of water (Scheme 3, path II).

To establish the actual mechanism and to prove the intermediate formation of bicyclo[3.2.1]octane derivatives **3** we carried out the reaction of specially prepared 6-imino-2,7-dioxabicyclo[3.2.1]octane-3,3,4-tricarbonitrile **3h** [21] with dry trifluoroacetic acid under anhydrous conditions (Table 2, entry 6). The successful implementation of this process and isolation of product **2h** with good yield indicate the path of transformations without the participation of water (Scheme 3, path II) as actual.

**Table 2:** Acid-catalysts screening^a^.

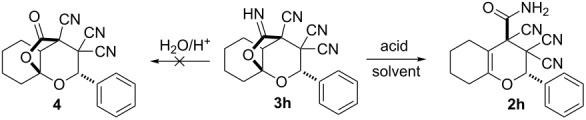					

Entry	Acid	Co-solvent	Conditions	Time (min)	Yield after 2 h^b^ (%)

1	HCl (aq 15%)	iPrOH	80 °C	5	71
2	HBr (aq 40%)	iPrOH	80 °C	3	65
3	HNO_3_ (aq 50%)	–	80 °C	3	67
4	H_2_SO_4_ (aq 50%)	1,4-dioxane	rt	15	–^c^
5	CH_3_COOH (dry)	–	110 °C	5	–^d^
6	CF_3_COOH (dry)	–	rt	10	69

^a^Reaction conditions: **3h** (0.5 mmol), acid (0.75 mL), solvent (0.75 mL, if marked); ^b^Isolated yield; ^c^The reaction does not stop at the amide formation, further transformations proceeds. ^d^The results were published previously [23].

Such an abnormal resistance of the imine moiety to be simply hydrolyzed in aqueous acidic media is very exciting. Therefore we attempted to use other acids to investigate the behavior of the imine moiety in presence thereof, but all acids promote only the iminolactone decyclization process without traces of lactone derivative **4** (Table 2).

It is also important to note that during the reaction pathway (Scheme 3) a carbonyl-assisted carbonitrile hydration effect (CACHE) was occured. The carbonyl group, through the formation of hemiketal (**A**), became a source of a hydroxy group that cyclized regiospecifically to the spatially proximate cyano group and caused the carboxamide formation. CACHE processes are essential for the chemistry of oxonitriles and sometimes can be the reason for unusual quasi-hydrolysis of the cyano group under mild conditions [15,19,23], but it was not mentioned by many authors [28–31].

## Conclusion

In conclusion, we have developed a new efficient approach to a rare group of heterocycles, namely, functionalized 3,4-dihydro-2*H*-pyran-4-carboxamides with variable frame and exceptional diastereoselectivity based on the reaction of available 4-oxoalkane-1,1,2,2-tetracarbonitriles (adducts of TCNE and ketones) with aldehydes in acidic media. Unusual processes of regiospecific quasi-hydrolysis of a cyano group and abnormal resistance of the imine moiety to be hydrolyzed in aqueous acidic media were observed in the course of the described transformation. Moreover, the intermediate 6-imino-2,7-dioxabicyclo[3.2.1]octane-4,4,5-tricarbonitriles **3** had demonstrated a cytotoxic activity in various cancer cell lines [32], therefore the derived 3,4-dihydro-2*H*-pyrans **2** are very promising for biological studies.

## Supporting Information

File 1 Experimental data and characterization of all new compounds.
