# Supplementary material for: Diastereoselective synthesis of 3,4-dihydro-2H-pyran-4-carboxamides through an unusual regiospecific quasi-hydrolysis of a cyano group
Source: Beilstein J Org Chem. 2016 Sep 27;12:2093–8. doi: 10.3762/bjoc.12.198 (PMC5082461; doi:10.3762/bjoc.12.198)
Supplement: File 1 — Experimental data and characterization of all new compounds. [file Beilstein_J_Org_Chem-12-2093-s001.pdf]

Supporting Information

for

Diastereoselective synthesis of 3,4-dihydro-2*H*-pyran-4-carboxamides through an unusual regiospecific quasi-hydrolysis of a cyano group

Mikhail Yu. Ievlev *[1], Oleg V. Ershov [1], Mikhail Yu. Belikov[1], Angelina G. Milovidova[1], Viktor A. Tafeenko[2] and Oleg E. Nasakin[1]

Address: [1]Department of Organic and Pharmaceutical Chemistry, Ulyanov Chuvash State University, Moskovskiy av. 15, Cheboksary, Russia and [2]Department of General Chemistry, Lomonosov Moscow State University, Leninskie gory 1, Moscow, Russia

Email: Mikhail Yu. Ievlev - hiliam@bk.ru

*Corresponding author

Experimental data and characterization of all new compounds

Table of contents

General remarks S2

Experimental section S3

NMR spectra of the products S6

X-ray structure S12

General remarks

The progress of reactions and purity of products were monitored by TLC on Sorbfil plates (spots were visualized under UV light, by treatment with iodine vapor, or by heating). Melting and decomposition points were determined on device Optimelt MPA100. IR spectra were recorded on an FSM-1202 spectrometer with Fourier transform from samples dispersed in nujol. The NMR spectra of were measured in DMSO-d6 on Bruker DRX-500 (operating frequencies 500 MHz and 125 MHz for ^{1}H and ^{13}C NMR respectively) spectrometers using TMS as an internal reference. The mass spectra were obtained on a Bruker Ultraflex MALDI-TOF mass spectrometer. The X-ray data was collected by using STOE diffractometer Pilatus100K detector, focusing mirror collimation Cu Kα (1.54086 Å) radiation, rotation method mode. STOE X-AREA software was used for cells refinement and data reduction. Data collection and image processing was performed with X-Area 1.67 (STOE & Cie GmbH, Darmstadt, Germany, 2013). Intensity data were scaled with LANA (part of X-Area) in order to minimize differences of intensities of symmetry-equivalent reflections (multi-scan method). The structures were solved and refined with SHELX program. The non-hydrogen atoms were refined by using the anisotropic full matrix least-square procedure. The final R = 0.036. Molecular geometry calculations were performed with the SHELX program, and the molecular graphics were prepared by using DIAMOND software.

Starting materials and solvents were obtained from ACROS ORGANICS™ and were used without further purification. The precursors 4-oxoalkane-1,1,2,2-tetracarbonitriles **1** were obtained by a general method.[1] Liquid aldehydes were purified by distillation before use, whereas solid ones were used without additional purification.

---

[1] Ievlev, M.Yu.; Ershov, O.V.; Belikov, M.Yu.; Lipin, K.V.; Fedoseev, S.V.; Nasakin, O.E. (2016). *Method for producing 4-oxoalkane-1,1,2,2-tetracarbonitriles*, Patent RU 2577537.

Experimental section

Typical procedure for the preparation of 3,3,4-tricyano-3,4-dihydro-2*H*-pyran-4-carboxamides 2a-j. To the suspension of appropriate 4-oxoalkane-1,1,2,2-tetracarbonitrile **1** (5 mmol) in the mixture of propan-2-ol (25 ml) and water (0.5 ml) a slight excess of appropriate aldehyde (5.1 mmol) was added. The mixture was stirred at room temperature until the starting compound **1** was completely disappeared (TLC). Then, 15% water solution of hydrochloric acid was carefully added and stirring continued while heating. After the complete dissolution of solid heating was stopped and mixture allowed to cool continuing stirring at room temperature. To initiante crystallization sometimes rubbing with a glass rod or seeding is necessary. Precipitated solid product was filtered, washed with an ice-cooled mixture of propan-2-ol and water (1:1) and allowed to dry in a vacuum desiccator over $CaCl_2$.

3,3,4-Tricyano-5,6-dimethyl-2-phenyl-3,4-dihydro-2H-pyran-4-carboxamide ***2a***. Mp 166-168°C (dec.); IR ν_{max}/cm^{-1} (nujol): 1711 (C=O), 2244 (C≡N), 3198, 3379 (N–H); ^1H NMR (500.13 MHz, DMSO-d$_6$): δ 1.90 (3H, s, CH_3), 2.02 (3H, s, CH_3), 5.90 (1H, s, OCH), 7.57 (5H, s, C_6H_5), 8.42 (1H, s, C(O)NH_2), 8.64 (1H, s, C(O)NH_2) ppm; HRMS calcd for $C_{17}H_{15}N_4O_2$ $[M+H]^+$: 307.1190, found: 307.1187.

3,3,4-Tricyano-5-(4-methoxyphenyl)-6-methyl-2-phenyl-3,4-dihydro-2H-pyran-4-carboxamide ***2b***. Mp 191-193°C (dec.); IR ν_{max}/cm^{-1} (nujol): 1719 (C=O), 2248 (C≡N), 3204, 3348 (N–H). ^1H NMR (500.13 MHz, DMSO-d$_6$): δ 1.79 (3H, s, CH_3), 3.81 (3H, s, OCH_3), 6.15 (1H, s, OCH), 7.06 (2H, d, *J*=8.7 Hz, $CH_3OC_6\underline{H}_4$), 7.20 (2H, d, *J*=8.7 Hz, $CH_3OC_6\underline{H}_4$), 7.50-7.68 (5H, m, C_6H_5), 8.27 (1H, s, C(O)NH_2), 8.68 (1H, s, C(O)NH_2) ppm; HRMS calcd for $C_{23}H_{19}N_4O_3$ $[M+H]^+$: 399.1452, found: 399.1449.

3,3,4-Tricyano-2-isopropyl-5-methyl-6-phenyl-3,4-dihydro-2H-pyran-4-carboxamide ***2c***. Mp 177-179°C (dec.); IR ν_{max}/cm^{-1} (nujol): 1709 (C=O), 2253 (C≡N), 3186, 3347 (N–H); ^1H NMR (500.13 MHz, DMSO-d$_6$): δ 1.15 (3H, d, *J*=6.8 Hz, $C\underline{H}_3CH$), 1.22 (3H, d, *J*=6.8 Hz, $C\underline{H}_3CH$), 1.90 (3H, s, CH_3), 2.32-2.39 (1H, m, $(CH_3)_2C\underline{H}$), 4.83 (1H, d, *J*=4.2 Hz, $OC\underline{H}CH$),

7.47-7.52 (5H, m, C_6H_5), 8.44 (1H, s, $C(O)NH_2$), 8.64 (1H, s, $C(O)NH_2$) ppm; HRMS calcd for $C_{23}H_{19}N_4O_3$ $[M+H]^+$: 399.1452, found: 399.1449.

3,3,4-Tricyano-2-isopropyl-6-(4-chlorophenyl)-5-methyl-3,4-dihydro-2H-pyran-4-carboxamide ***2d***. Mp 202-203°C (dec.); IR ν_{max}/cm^{-1} (nujol): 1712 (C=O), 2251 (C≡N), 3149, 3348 (N–H); ^1H NMR (500.13 MHz, DMSO-d$_6$): δ 1.15 (3H, d, *J*=6.8 Hz, C$\underline{H}_3$CH), 1.21 (3H, d, *J*=6.8 Hz, C$\underline{H}_3$CH), 1.90 (3H, s, CH_3), 2.32-2.39 (1H, m, $(CH_3)_2C\underline{H}$), 4.80 (1H, d, *J*=4.1 Hz, OC$\underline{H}$CH), 7.48-7.57 (4H, m, ClC_6H_4), 8.44 (1H, s, $C(O)NH_2$), 8.65 (1H, s, $C(O)NH_2$) ppm; HRMS calcd for $C_{19}H_{18}ClN_4O_2$ $[M+H]^+$: 369.1113, found: 369.1110.

3,3,4-Tricyano-2-methyl-3,4,5,6,7,8-hexahydro-2H-chromene-4-carboxamide ***2e***. Mp 151-152°C (dec.); IR ν_{max}/cm^{-1} (nujol): 1706 (C=O), 2255 (C≡N), 3192, 3376 ($C(O)NH_2$); ^1H NMR (500.13 MHz, DMSO-d$_6$): δ 1.53-1.58 (2H, m, CH_2), 1.60 (3H, d, *J*=6.2 Hz, CH_3), 1.69-1.76 (2H, m, CH_2), 2.03-2.08 (1H, m, CH_2), 2.14-2.19 (3H, m, 2CH_2), 4.91 (1H, q, *J*=6.2 Hz, 18.6, OCH), 8.26 (1H, s, $C(O)NH_2$), 8.53 (1H, s, $C(O)NH_2$) ppm; HRMS calcd for $C_{14}H_{15}N_4O_2$ $[M+H]^+$: 271.1190, found: 277.1188.

3,3,4-Tricyano-2-isobutyl-3,4,5,6,7,8-hexahydro-2H-chromene-4-carboxamide ***2f***. M.p. 128-130°C (dec.); IR ν_{max}/cm^{-1} (nujol): 1707 (C=O), 2259 (C≡N), 3204, 3376 (N–H); ^1H NMR (500.13 MHz, DMSO-d$_6$): δ 0.94 (3H, d, *J*=6.6 Hz, CH_3), 1.00 (3H, d, *J*=6.7 Hz, CH_3), 1.51–1.63 (3H, m, 2CH_2), 1.67–1.78 (3H, m, 2CH_2), 1.79–1.86 (1H, m, CH), 1.89–1.96 (1H, m, CH_2), 2.00–2.07 (1H, m, CH_2), 2.12–2.19 (2H, m CH_2), 4.80 (1H, dd, *J*=10.3, 2.3, OCH), 8.31 (1H, s, $C(O)NH_2$), 8.52 (1H, s, $C(O)NH_2$) ppm; HRMS calcd for $C_{17}H_{21}N_4O_2$ $[M+H]^+$: 313.1659, found: 313.1657.

3,3,4-Tricyano-2-cyclohexyl-3,4,5,6,7,8-hexahydro-2H-chromene-4-carboxamide ***2g***. Mp 149-151°C (dec.); IR ν_{max}/cm^{-1} (nujol): 1721 (C=O), 2257 (C≡N), 3181, 3471 (N–H); ^1H NMR (500.13 MHz, DMSO-d$_6$): δ 1.10–1.42 (5H, m, 3CH_2), 1.52–1.82 (8H, m, 4CH_2), 1.92–2.01 (2H, m, CH_2), 2.04–2.23 (4H, m, 2CH_2), 4.58 (1H, a, *J*=3.6 Hz, OCH), 8.22 (1H, s, $CONH_2$), 8.48 (1H, s, $CONH_2$) ppm. HRMS calcd for $C_{19}H_{23}N_4O_2$ $[M+H]^+$: 339.1816, found: 339.1814.

3,3,4-Tricyano-2-phenyl-3,4,5,6,7,8-hexahydro-2H-chromene-4-carboxamide ***2h***. Mp 186-187°C (dec.); IR ν_{max}/cm^{-1} (nujol): 1708 (C=O), 2244 (C≡N), 3184, 3413 (N–H); ^{1}H NMR (500.13 MHz, DMSO-d$_6$): δ 1.54-1.69 (2H, m, CH$_2$), 1.75-1.83 (2H, m CH$_2$), 2.15-2.37 (4H, m, 2CH$_2$), 5.95 (1H, s, OCH), 7.57 (5H, s, C$_6$H$_5$), 8.42 (1H, s, C(O)NH$_2$), 8.67 (1H, s, C(O)NH$_2$) ppm; ^{13}C NMR (125 MHz, DMSO-d$_6$): δ 21.3, 21.9, 23.3, 24.4, 43.0, 53.5, 74.4, 102.0, 113.0, 113.3, 117.2, 128.5 (2C), 129.7, 129.9, 133.2, 134.1, 152.0, 167.6 ppm; HRMS calcd for $C_{19}H_{17}N_4O_2$ [M+H]$^+$: 333.1346, found: 333.1343.

2-(2-Bromophenyl)-3,3,4-tricyano-3,4,5,6,7,8-hexahydro-2H-chromene-4-carboxamide ***2i.*** Mp 197-198°C (dec.); IR ν_{max}/cm^{-1} (nujol): 1711 (C=O), 2253 (C≡N), 3187, 3482 (N–H). ^{1}H NMR (500.13 MHz, DMSO-d$_6$): δ 1.55-1.68 (2H, m, CH$_2$), 1.71-1.86 (2H, m, CH$_2$), 2.01-2.17 (2H, m, CH$_2$), 2.19-2.31 (2H, m, CH$_2$), 6.31 (1H, s, OCH), 7.54 (1H, t, *J*=7.7 Hz, C$_6$H$_4$Br), 7.66 (1H, t, *J*=7.6 Hz, C$_6$H$_4$Br), 7.78 (1H, d, *J*=7.8 Hz, C$_6$H$_4$Br), 7.84 (1H, d, *J*=7.8 Hz, C$_6$H$_4$Br), 8.44 (1H, s, C(O)NH$_2$); 8.70 (1H, s, C(O)NH$_2$) ppm. HRMS calcd for $C_{19}H_{16}BrN_4O_2$ [M+H]$^+$: 411.0451, found: 411.0449.

3,3,4-tricyano-2-phenyl-2,3,4,5,6,7,8,9-octahydrocyclohepta-[b]pyran-4-carboxamide ***2j.*** Mp 171-173°C (dec.); IR ν_{max}/cm^{-1} (nujol): 1708 (C=O), 2256 (C≡N), 3183, 3417 (N–H); ^{1}H NMR (500.13 MHz, DMSO-d$_6$): δ 1.41-1.52 (2H, m, CH$_2$); 1.61-1.71 (3H, m, 2CH$_2$); 1.81-1.88 (1H, m, CH$_2$); 2.35-2.42 (3H, m, 2CH$_2$); 2.50-2.59 (1H, m, CH$_2$); 5.92 (1H, s, OCH); 7.57 (5H, s, C$_6$H$_5$); 8.40 (1H, s, C(O)NH$_2$); 8.62 (1H, s, C(O)NH$_2$) ppm; ^{13}C NMR (125 MHz, DMSO-d$_6$): δ 24.3, 25.9, 28.4, 30.7, 32.9, 44.8, 54.2, 74.1, 99.9, 110.7, 110.9, 115.4, 127.4 (2C), 128.9 (2C), 130.8, 132.3, 158.6, 163.0 ppm; HRMS calcd for $C_{20}H_{19}N_4O_2$ [M+H]$^+$: 347.1503, found: 347.1501.

NMR spectra of the products

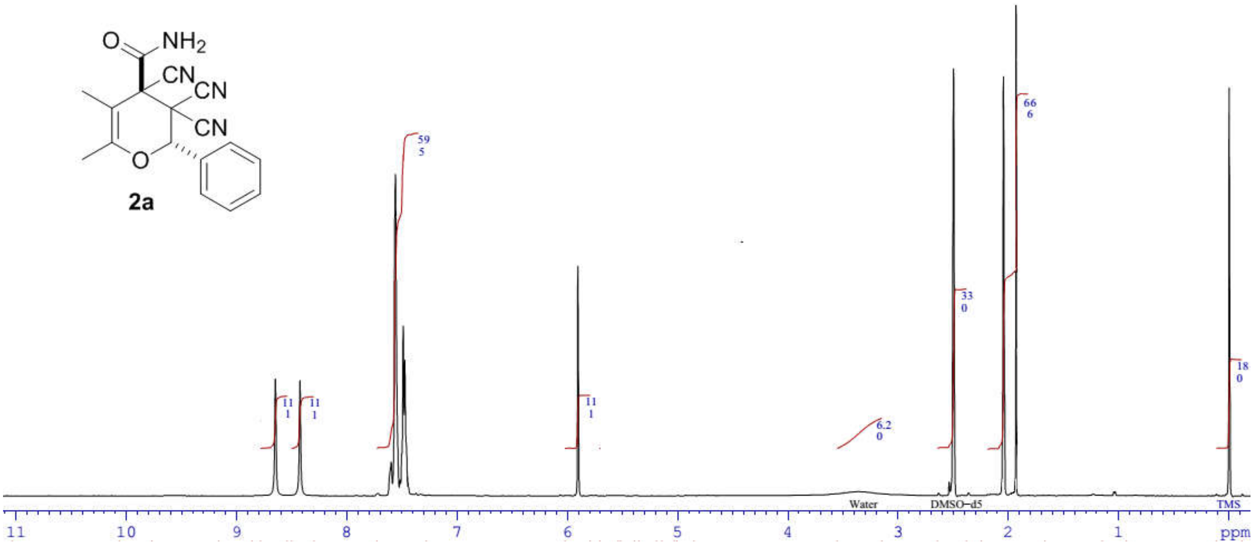

^{1}H-NMR-spectrum (500 MHz, DMSO d6, 299K) of **2a**

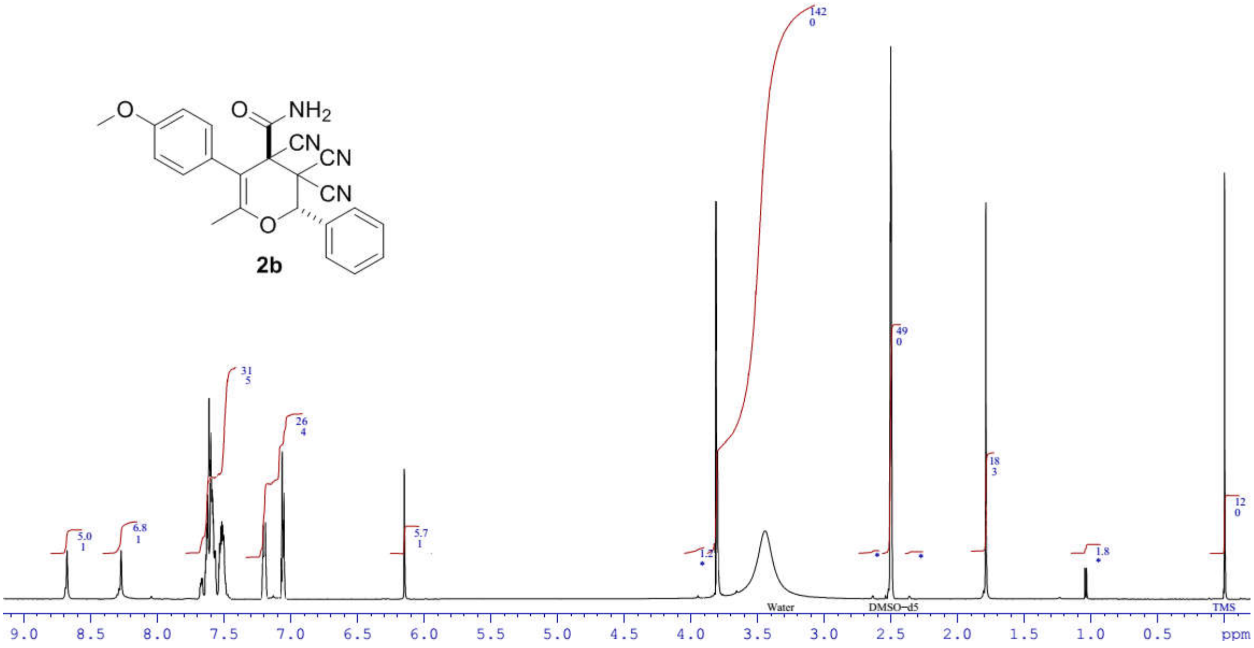

^{1}H-NMR-spectrum (500 MHz, DMSO d6, 299K) of **2b**

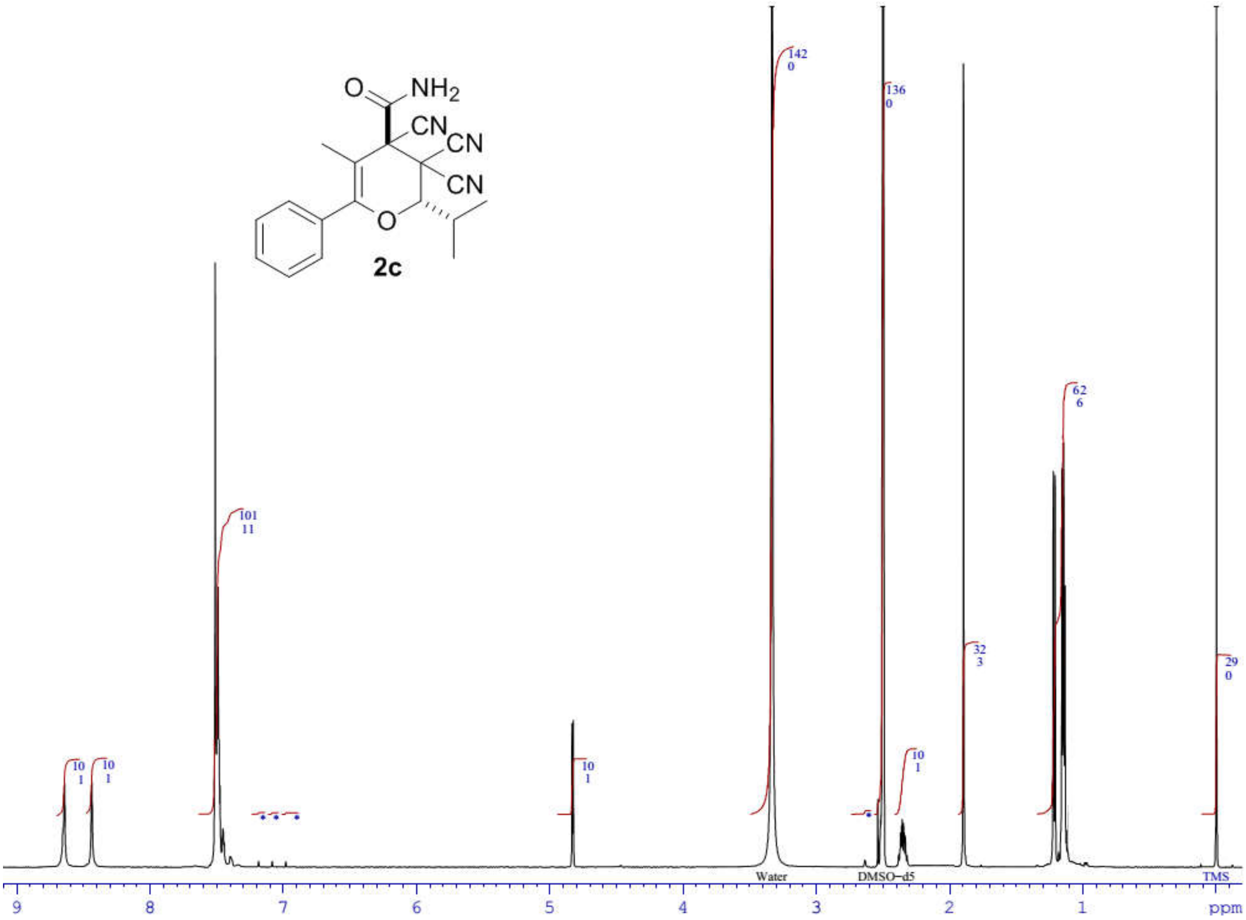

^{1}H-NMR-spectrum (500 MHz, DMSO d6, 299K) of **2c**

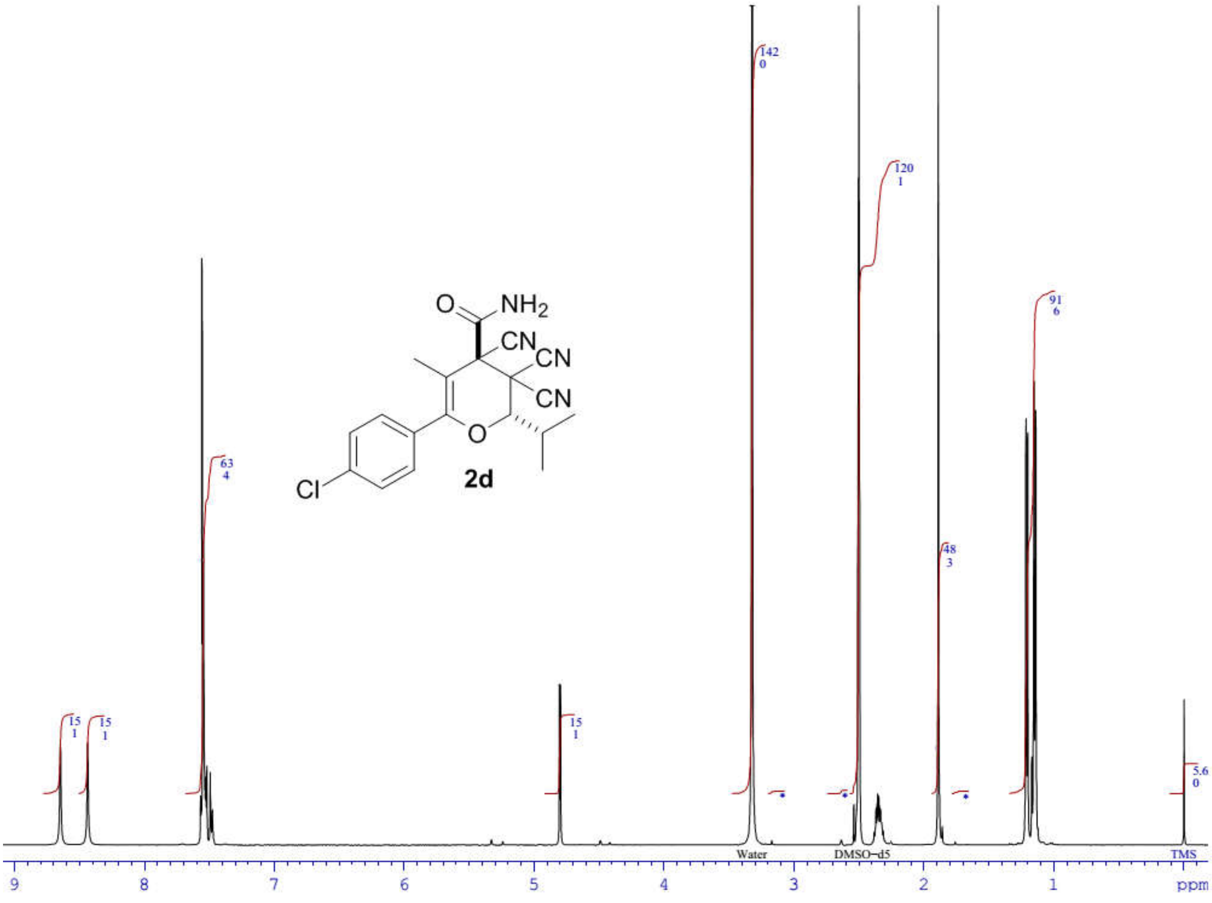

^{1}H-NMR-spectrum (500 MHz, DMSO d6, 299K) of **2d**

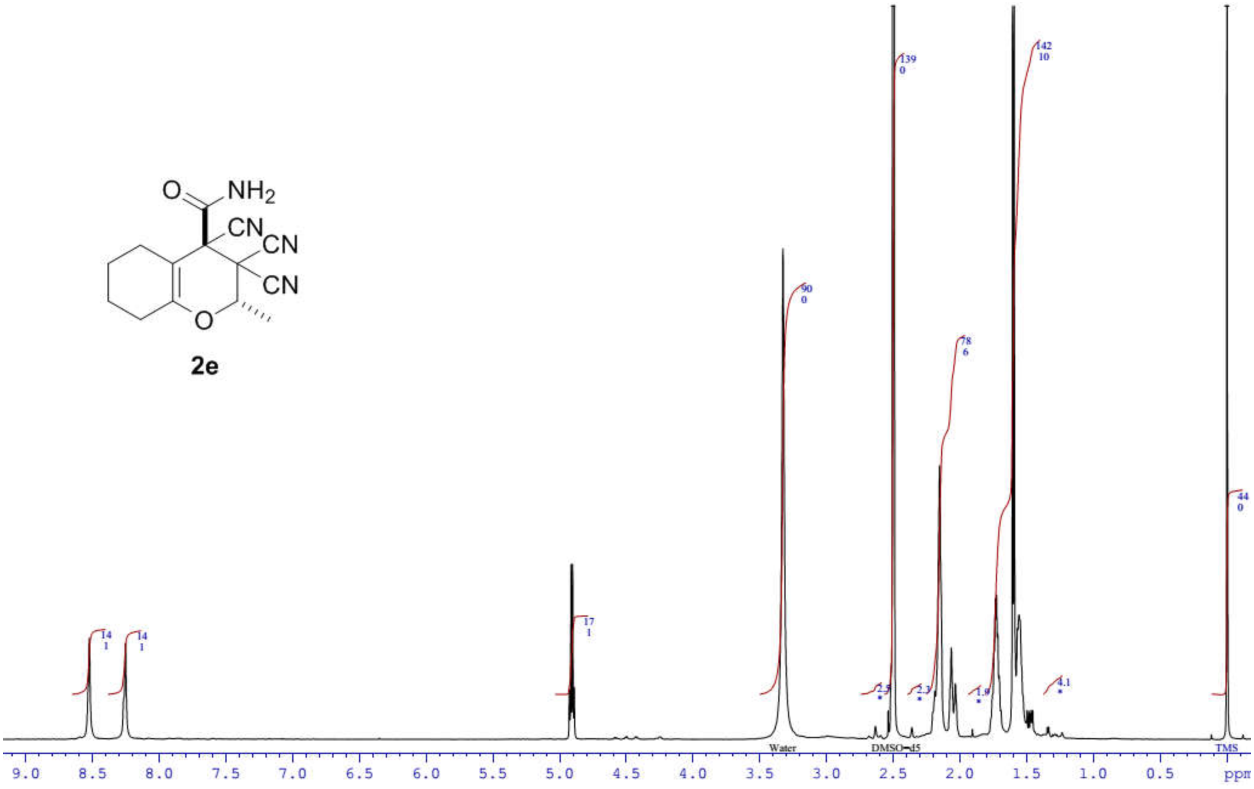

^{1}H-NMR-spectrum (500 MHz, DMSO d6, 299K) of **2e**

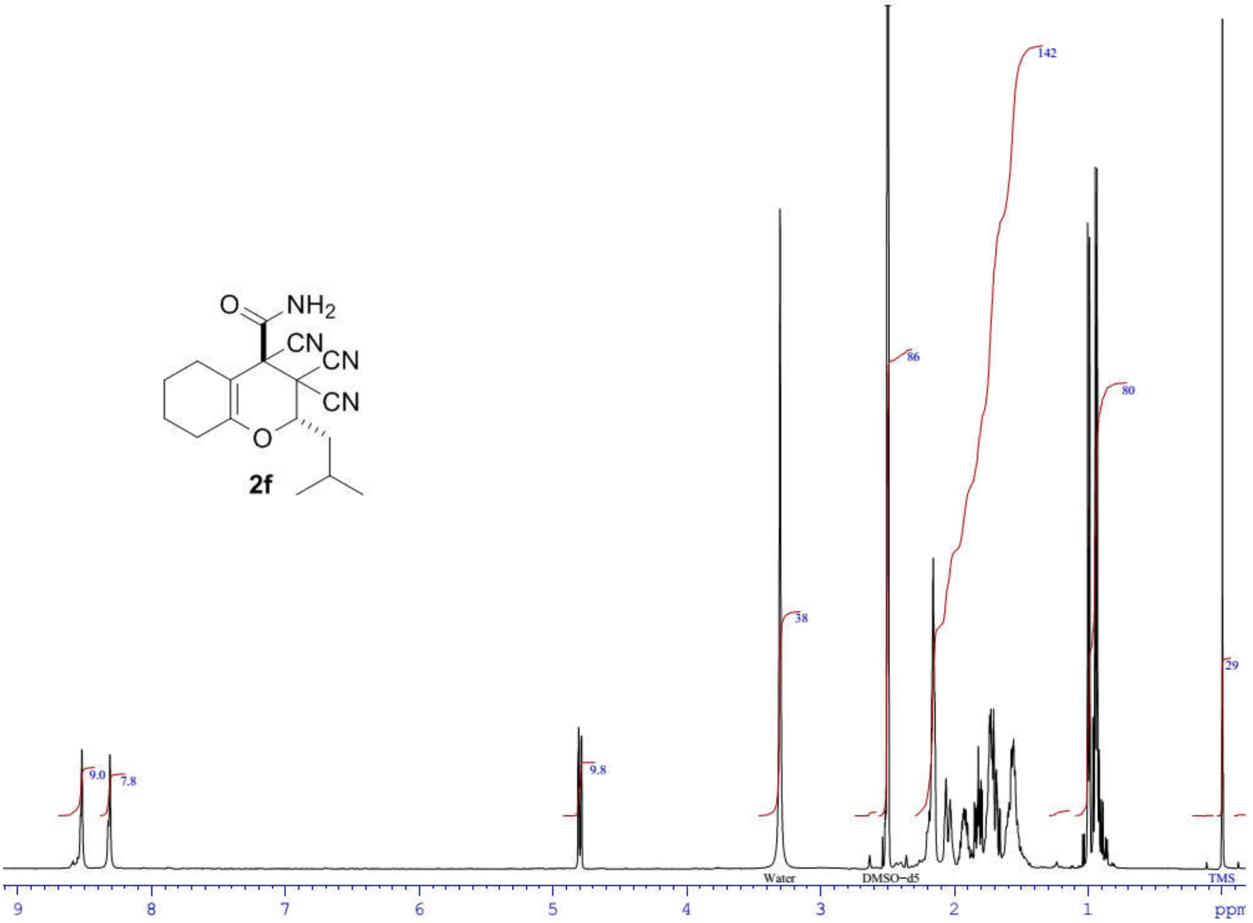

^{1}H-NMR-spectrum (500 MHz, DMSO d6, 299K) of **2f**

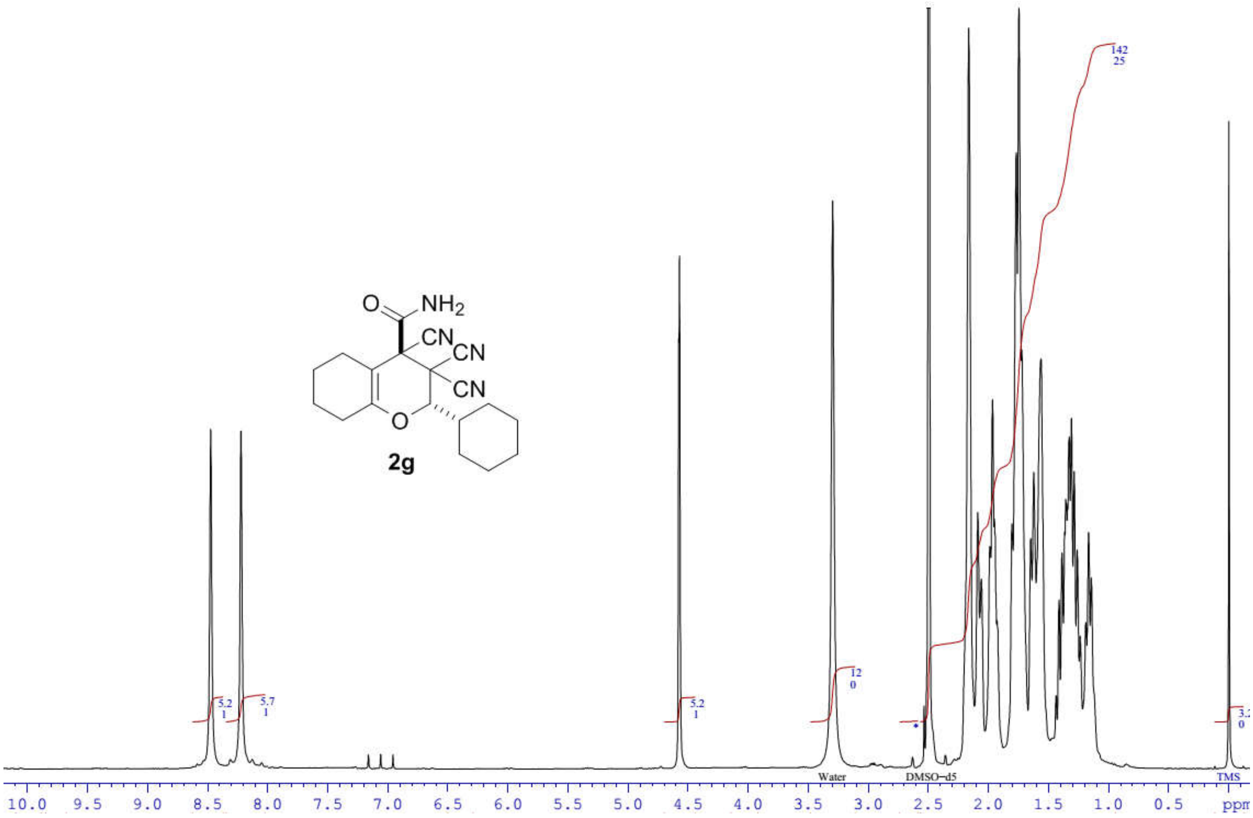

^{1}H-NMR-spectrum (500 MHz, DMSO d6, 299K) of **2g**

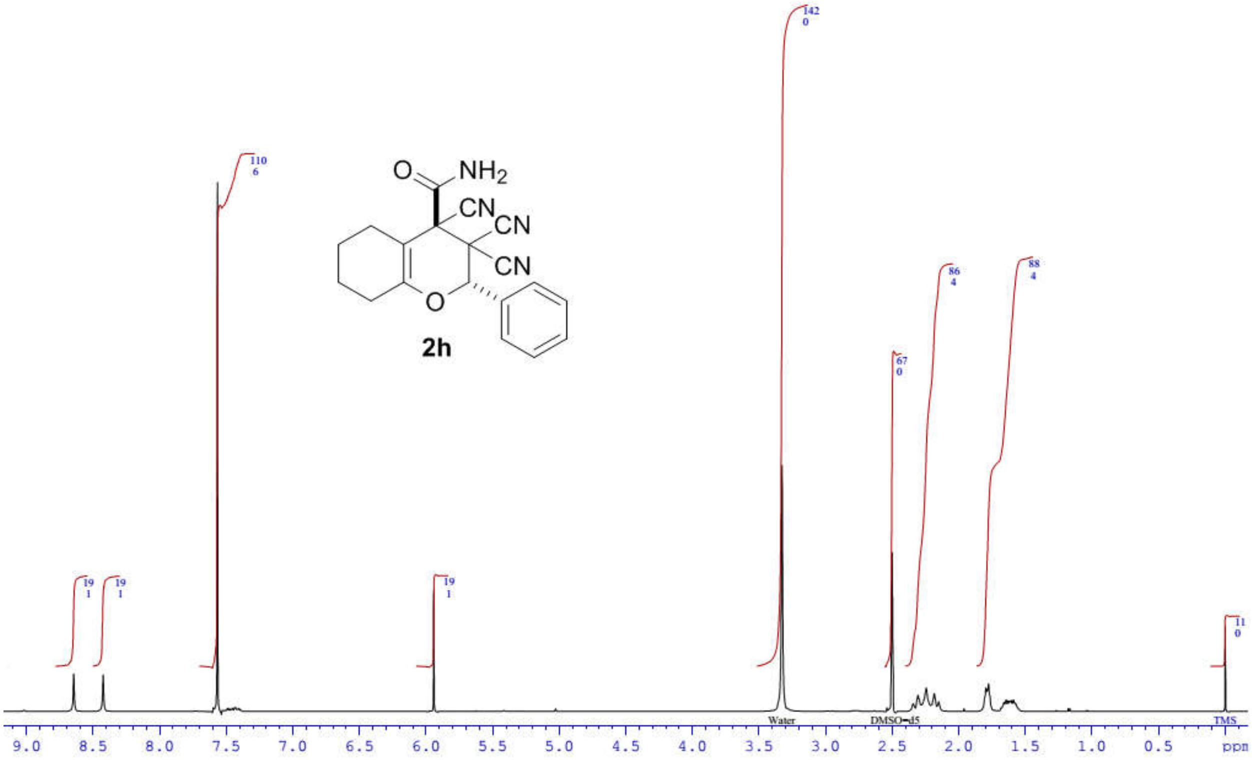

^{1}H-NMR-spectrum (500 MHz, DMSO d6, 299K) of **2h**

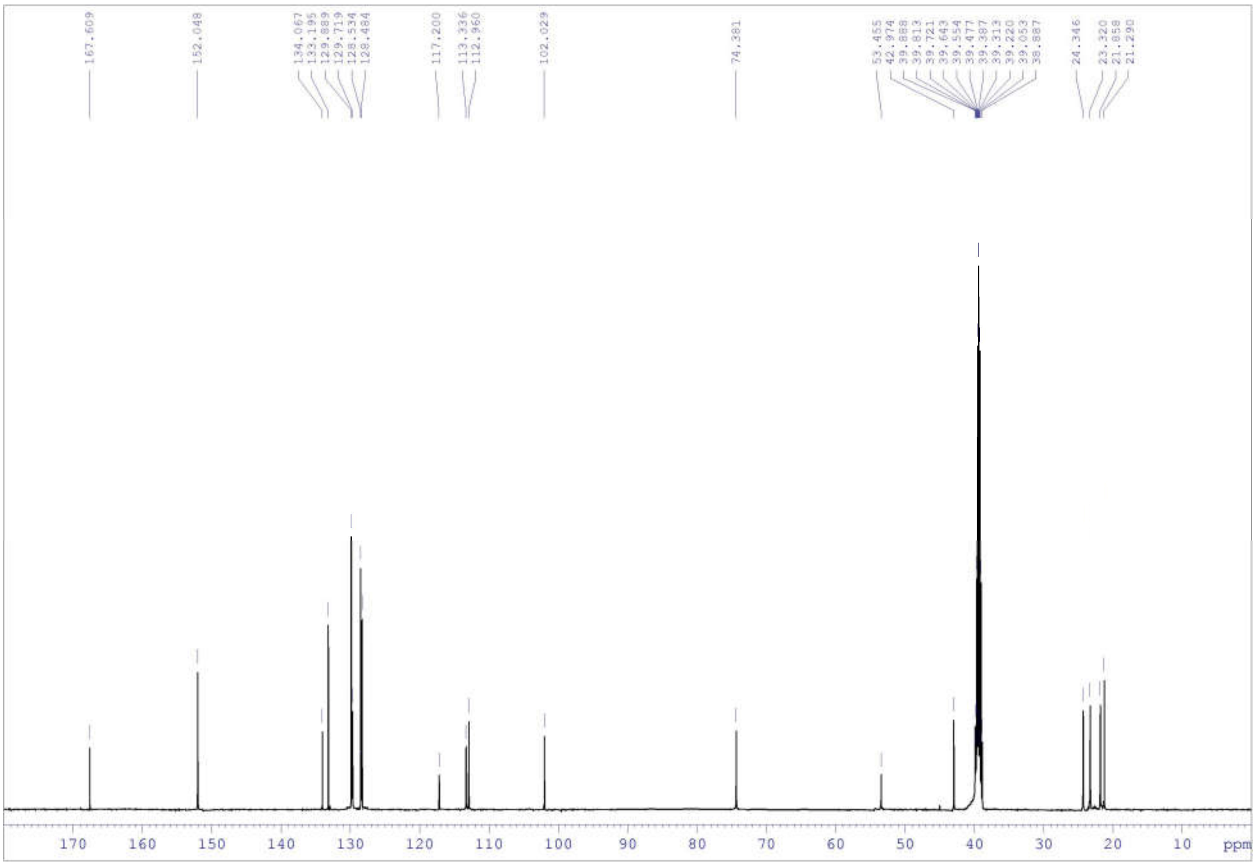

^{13}C-NMR-spectrum (125 MHz, DMSO d6, 299K) of **2h**

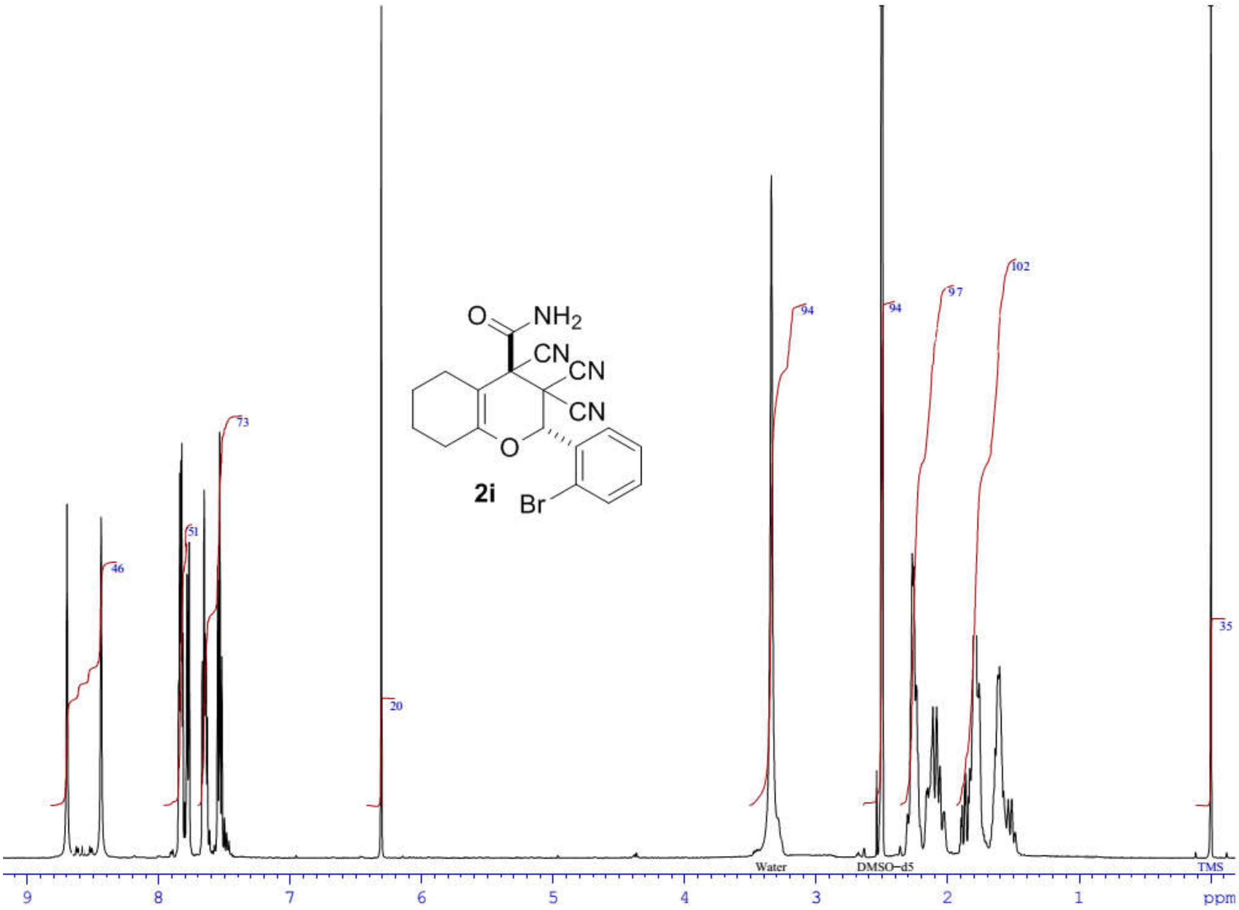

^{1}H-NMR-spectrum (500 MHz, DMSO d6, 299K) of **2i**

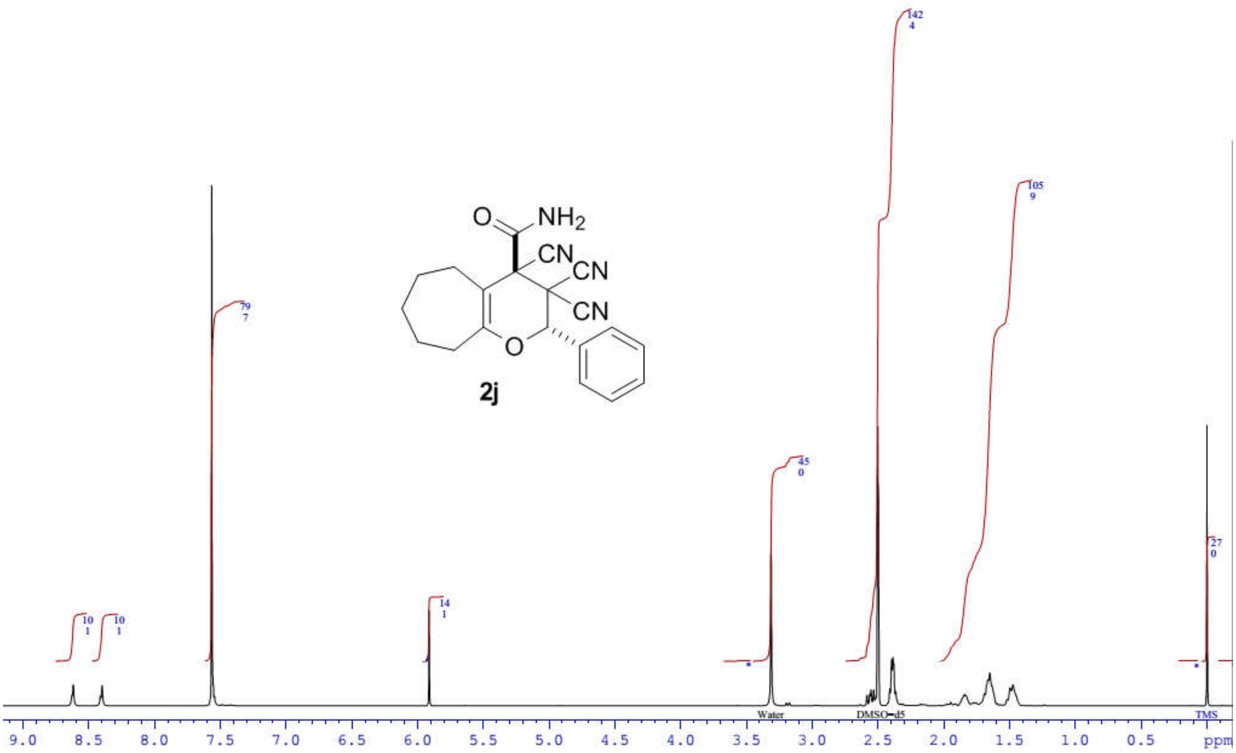

^{1}H-NMR-spectrum (500 MHz, DMSO d6, 299K) of **2j**

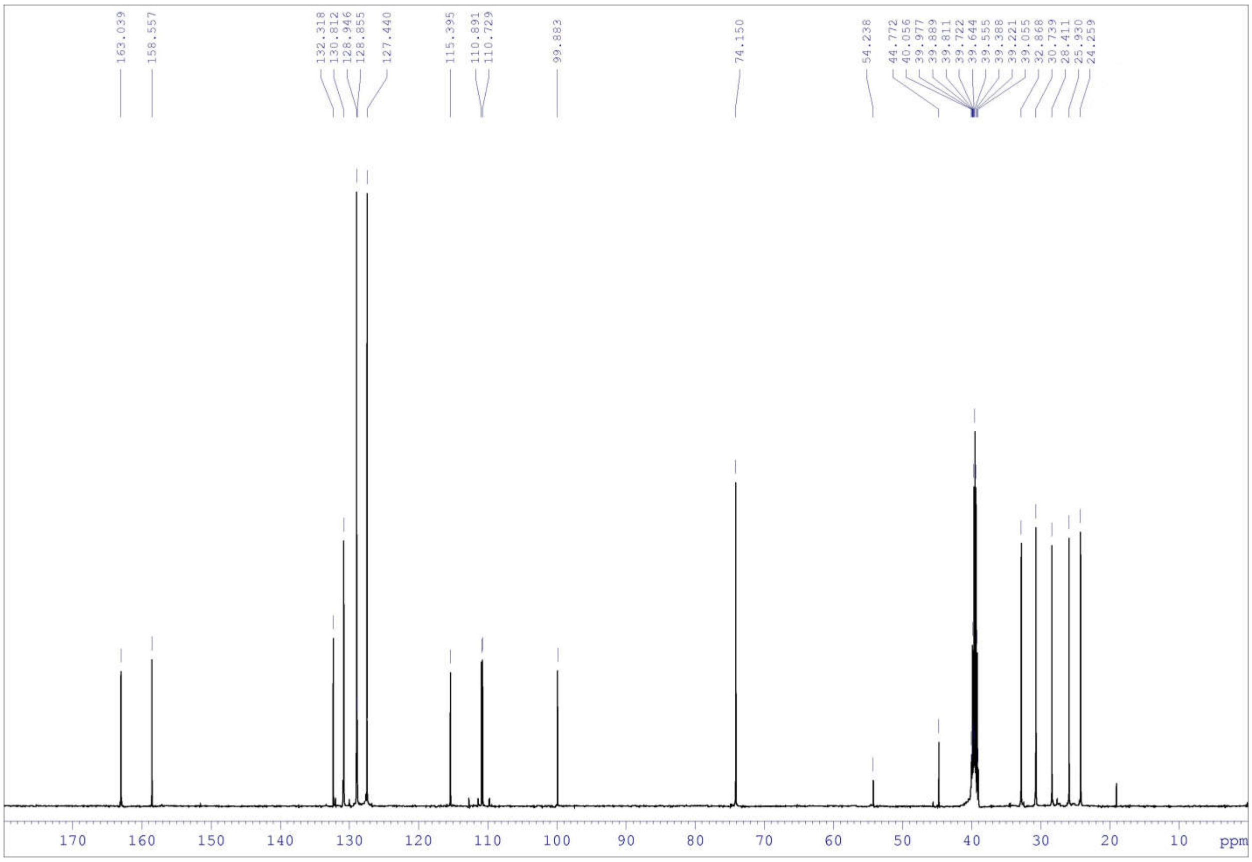

^{13}C-NMR-spectrum (125 MHz, DMSO d6, 299K) of **2j**

X-ray structure

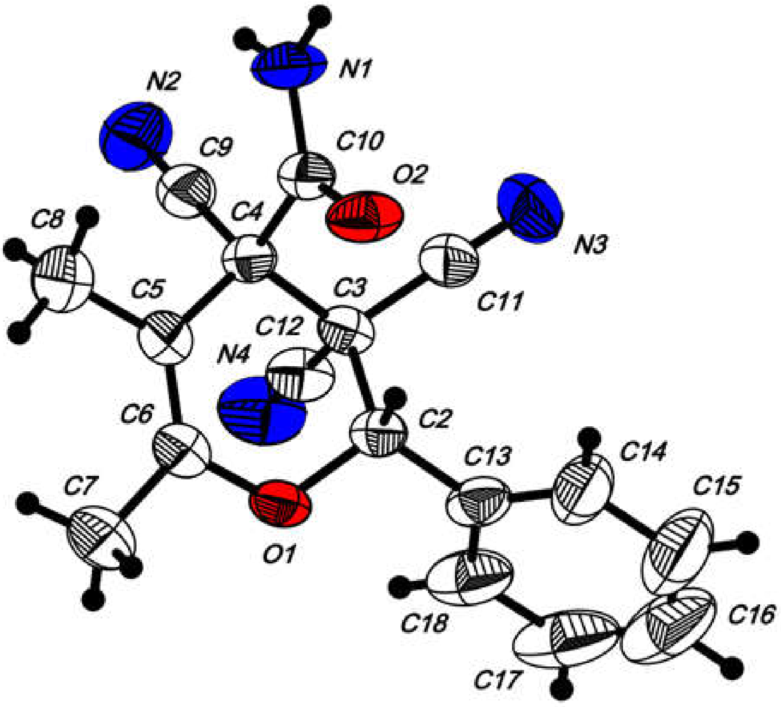

The molecular structure of **2a** with the atom-numbering scheme. Displacement ellipsoids are drawn at the 50% probability level and H atoms are drawn as small spheres of arbitrary radii.
